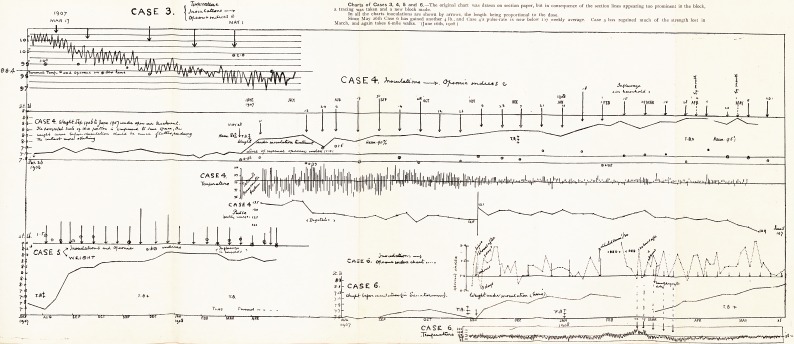# Is Opsonic Treatment Useful in Phthisis?
1Read before the Bristol and Bath Branch of the British Medical Association at Bath on April 29th, 1908.


**Published:** 1908-06

**Authors:** John M. H. Munro


The Bristol

Medico-Chirurgical Journal.

*"Scire est nescire, nisi id me
Scire alius sciret."*

JUNE, 1908.

### IS OPSONIC TREATMENT USEFUL IN PHTHISIS?[1]

BY

JOHN M. H. MUNRO, D.Sc. Lond.

THIS question, often asked by interested patients and their relatives, is still met by the majority of family doctors with an answer in the negative, and an appeal to a consultant does not usually result in any material alteration in the advice given.

Although the object of this paper is to incline readers to an opposite verdict, one cannot complain of the adoption of a cautious attitude. Those old enough in practice to remember the sequence of events on the introduction of Koch's original tuberculin, and those young enough to be impressed with the definite results often following the administration of single doses of vaccines in the infinitesimal amounts now used, are both readily justified in this attitude. Tuberculin, if used at all, must not be placed on the same level with a new drug or " combination " recommended to our notice by the manufacturing chemists. The recognition

[1] Read before the Bristol and Bath Branch of the British Medical Association at Bath on April 29th, 1908.

of this renders it necessary to distinguish between tuberculin treatment and opsonic treatment.

There are three points to note about the failure of tuberculin on its first introduction. The original preparation did not consist of the whole bacillary substance in as slightly altered a form as possible, and hence did not conform in composition with modern vaccines, which are administered with the definite object of stimulating the production of protective substances in the blood. Next, being apparently given with the idea that if the patient could be got to stand a sufficient amount of it the disease would be vanquished, the natural anxiety of doctor and patient to attain this point led to pushing the administration of the remedy to a dangerous extent. Much the same thing happened in the history of the use of mercury in syphilis, and the result was similar. Mercury was at one time discredited for many years amongst a considerable section of the medical profession, and tuberculin was, after a few years' extensive trial, put on the shelf except by a mere handful of practitioners. The third point is that clinical observation alone did not suffice to reform the administration of tuberculin, but led rather to its general abandonment. Its reinstatement as an agent of great therapeutic value has been brought about partly by the laborious researches of the last few years into the formation of anti-bodies in the blood, and their rôle in the production of immunity to bacterial infections; partly by the efforts of Sir Almroth E. Wright, who had never given up the use of tuberculin, to control inoculations by devising means of testing their effects without relying on clinical observation alone. These efforts naturally took the form of attempts to measure the anti-bacterial or protective substances in the blood, whether of normal persons, of those suffering from bacterial diseases, or of the subjects of prophylactic or curative inoculations. These substances being of various descriptions—lysins, agglutinins, precipitins, bactericidal substances, &c.—their production can sometimes be followed by more than one test.

Wright's efforts to measure the effects of individual inoculations of tuberculin by estimating the increase in the agglutinating power of the blood serum for the tubercle bacillus, had already, in conjunction with his researches on other bacterial vaccines, guided him, before his discovery of opsonins, to a more rational use of tuberculin, and had enabled him to make many striking cures of tubercular lesions in patients who had suffered from the older method of inoculation. When, in conjunction with Douglas, he revealed the existence in the blood of a hitherto unknown class of protective substances, Metchnikoff's insistence on the importance of phagocytosis in repelling bacterial invasions was soon placed in an unassailable position.

Briefly, phagocytosis—the ingestion of invading bacteria by leucocytes—is the common and generally the main defensive action of the organism. The leucocytes are powerless to effect this ingestion without the presence of opsonin in the blood, which opsonin is absorbed by the bacteria; and, further, the production of specific opsonins can be stimulated by the inoculation of suitable doses of bacterial vaccines.

Again, the opsonic power of the blood serum can be measured by Wright's method of determining the opsonic index, and it may be taken as definitely proved that this varies little in healthy persons, but undergoes great, and sometimes rapid, alteration in the subjects of infection. This is accounted for by the facts that opsonin is withdrawn from the blood in the process of phagocytosis, and is produced in response to the stimulation caused by the absorption of invading bacteria and their products into the blood stream. It is also produced by the inoculation of dead bacteria in the form of vaccines, and hence the effects of inoculation and of auto-inoculation are, at any rate in part, similar.

Opsonic treatment, therefore, has to take cognisance of the patient's condition as regards auto-inoculations, to either minimise them or utilise them when they take place, and to consider the desirability of supplementing or replacing them by inoculations of vaccine. To minimise them must always be the first effort, since large and irregular inoculations—auto or artificial—produce the result which it is the chief effort of the immuniser to avoid, viz. over-stimulation and a consequent prolonged fall in the production of protective substances. This is the so-called negative phase, during which an extension of the disease is favoured. Rest and a reduction in the coagulation time of the blood (to which end the giving of calcium salts may supplement the milk diet) are the main factors in minimising auto-inoculation. Having attained this end, massage, or regulated exercise, or injections of tuberculin, may be used to induce controlled stimulations of the protective machinery. If tuberculin is used, exercise and massage should be prohibited at first, in order that the effect of the inoculations may be studied as nearly as possible without complications, and when later graduated exercise is prescribed, it should be done so as to supplement and not interfere with the injections. Drugs which hinder phagocytosis should be avoided as routine treatment, but iron, in the anæmic cases, and strychnine may be given for long periods.

Further, it is an essential feature of vaccine therapy that the blood must not only be charged with protective substances, but must be brought to the focus of infection in increased quantity if possible. In dealing with gross lesions of the limbs and superficies, one can accomplish this by cupping or bandaging according to the method of Bier. In phthisical cases the same result is said to be achieved by restricting the intake of air during inspiration, whilst expiration is made as free as possible. This can be done by closing the mouth and one nostril during prolonged inspiration only, or by the use of Kuhn's suction mask. In two or three of the cases cited below, breathing exercises on this plan were prescribed, and were apparently useful.

The taking of the opsonic index is employed (1) to assist in diagnosis; (2) to ascertain the condition as regards auto-inoculation; (3) to control the effects of tuberculin injections, of exercise, and of massage; (4) to keep a watch on the patient's condition after an apparent cure: and will be found useful in proportion to the experience which the operator has had with it.

But even were a case treated on the above lines without the taking of a single index, it would be in a sense opsonic treatment, since without the many thousands of determinations made by Wright and his pupils the principles above enunciated could not have been laid down. These have shown conclusively that in giving tuberculin by the old method—large doses rapidly increased and at diminishing intervals—the opposite effects to the desired ones were certain to be produced.

These introductory remarks have been necessary to emphasise the difference between the ideas which govern the modern administration of tuberculin, and those which obtained on its first introduction, and we may hope that the remark, " Tuberculin was tried years ago, and did nothing but send up the temperature and aggravate the disease," will not be much longer used as a deterrent.

Another frequent reply to inquirers—" Tuberculin treatment is on its trial : we must wait until results on a large scale are available "—has an element of truth and caution, and I do not think anyone could be blamed for not advising tuberculin in phthisical cases whose previous experience with extra pulmonary tubercular lesions had not convinced him of its utility in a very decisive manner. My own conviction, based on the tubercular cases I saw in all stages of treatment at St. Mary's, that one could not employ tuberculin rationally in half a dozen successive cases of palpable tubercular lesions without encountering cogent evidence of its specific efficacy, was borne out in my own experience directly I was able to put it to the test.

One of the first cases was that of a man, H. T., with tubercular wrist, whom I showed at Bath in November 1906, half cured of a lesion involving the whole dorsum of the right hand and the ulnar side of the palm, discharging by three openings, with minute bone fragments occasionally coming away. For a year before treatment the hand, although at rest in a splint, had been getting worse and the pain increasing. Absolutely no change was made in the treatment, no medicine was administered, and there was no change of residence. The man came from Devizes to Bath at regular intervals to be inoculated, and has now for more than a year been using the hand in full work as a hedger, ditcher, and thatcher. Opsonic index before treatment 0.5, always higher since. Opsonic index of pus never higher than that of blood, generally lower, once or twice very low. There is no pain, all the movements are intact, and the cure is as nearly as possible perfect, although inoculations are still being given at long intervals, as under heavy work or a little indulgence a drop of serum still makes its way out at one minute point.

Or take one of my last cases. A young cook came to me with a tubercular ulceration affecting the terminal phalanx of a finger. This, under simple dressings with boracic lint and the exhibition of medicines which are often useful in such lesions, had failed to heal in twelve months. (A younger sister was attending hospital with tubercular abscess of leg.) The index at starting was 0.63, and I commenced inoculations with the intention of using Bier's treatment also if necessary. Two inoculations, however, healed the finger within twenty days, no medicine being given and the dressings not changed.

A strict *experimentum crucis* is hardly realisable in medicine, for when all material conditions have been arranged so that the only one to be varied at will is the therapeutic agent under test, one has still to reckon with uncontrollable changes in the patient, with psychical influences and the possibility of faith cures. But it will be admitted that the two cases cited are as nearly crucial as possible, and that although such striking results only follow in a percentage of cases, they thoroughly justify the routine employment of tuberculin as a specific remedy. Nor could such cases fail to stimulate one to try cautiously whether, at any rate, some cases of pulmonary tuberculosis could not be found to respond to tuberculin in a similar unmistakable manner.

Proceeding tentatively, and prepared to drop altogether the remedy unless unequivocal instances of benefit should present themselves, I found it was not necessary to treat half a dozen cases before encountering such instances. The first two or three yielded evidence of the kind required, and I have brought them forward with others in this paper in chronological order, so that the impression made on my own mind may be reproduced in the reader's.

Without waiting for statistical comparisons of batches of similar cases treated with and without tuberculin, or in any way appealing to the experience or opinion of others, I am able to decide for myself, on a basis of experience available to every private practitioner, that in dropping the use of tuberculin I should be depriving my phthisical patients of a remedy which may or may not determine or accelerate a cure in a given case, but cannot, so far as my knowledge extends, be replaced by any other having the same valid claim to specific action. The cases cited, although few in number, include almost all types of phthisis, recent and long-standing, acute and chronic, febrile and apyrexial, with and without hemorrhage, discoverable cavitation, or pleuritic complications, one and both lungs involved, etc. Some are apparently cured, and some may probably never be cured; but they all present the one feature essential to my argument—of certain definite and sometimes measurable improvements occurring under conditions approaching crucial ones, and therefore not admitting of probable explanation apart from the administration of tuberculin. The use which has been made of the opsonic index will appear in the notes of each case. The technique employed in its determination is that learned under Sir A. Wright, with the slight modification that I fix all my films by heat. The counting is done by my laboratory assistant, with no knowledge of the cases or circumstances. One hundred cells are counted in each slide, in two batches of fifty; these usually show close agreement, and the total is accepted. If not, another fifty are counted, and so on until it is considered the average is a fair one. Clumps, which are rare, are excluded from the counts. When one has in this way determined even a thousand indices, one knows the degree of accuracy to be expected from the method, its limitations, and most of the disturbing factors; and I am not aware that anyone with even this amount of personal experience has failed to obtain concordant results. To-day, for example, I sent down two samples of blood taken from the same patient at an interval of half an hour, the patient lying on a sofa meanwhile. They were marked I. and II. merely, and the indices returned were 2.20 and 2.21. The counts were: I.—First fifty leucocytes 80, second fifty 87, total 167; II.—First fifty 83, second fifty 85, total 168.

There are, of course, questions pending of the greatest interest with regard to the whole subject of opsonins and their estimation, but as they in no way affect the validity of any conclusions here made use of, which are simply the most general ones emerging from the observation of some hundreds of cases, and the determination of certainly over 30,000 indices by uniform methods by Wright and his pupils, I need not discuss them. In default of being able to measure all the factors in the production of immunity, it is surely of great advantage to be able to measure, though laboriously and imperfectly, one of the chief, and this the opsonic method enables us to do, as matters now stand, better than any other suggested means.

**Case 1.**—H. R., æt. 9. First seen August 17th, 1906. Sixteen months before, this boy was sent for treatment to dispensary, where signs of phthisis were found in right apex. Under treatment and away from school weight rose from 41 to 47 lb. in two months. Again attended in April of 1906, weighing only 47½ lb. After four months' treatment weighed 49 lb. Slight cough and expectoration. It was then proposed to send him to the Union Infirmary for open-air treatment, but at the suggestion of a guardian I undertook to treat him by inoculation at home. I thought and said that although the lung might heal, he was too much of a weed to fatten. He was too tired to play about with other boys, dulness at right apex, a little morning cough with T.B. fairly abundant in sputum. He was kept on same medicine as he got at the dispensary, and on August 24th first inoculation was given, his opsonic index at the time being 0.35. The cough ceased after this first injection, and no further specimen of sputum could be got until September 17th, when he coughed up a very small quantity in the morning in which no T.B. could be found. Inoculations were continued about every fourteen days. After the second he felt very well, and began to play; in two months he gained 3 lb. and weighed 52 lb., felt perfectly well, and went for long walks with his father. Although inoculations were continued for four months longer, he made no further gain in weight. The index was taken several times, and never was so low as before the first inoculation. Soon after this he attended school, and now sells newspapers in his spare time. He comes up to me now and then to say he is quite well, but he has not gained (May, 1908) more than 3 lb. since inoculations were discontinued. The crucial points in this case are the diagnosis made by another medical man sixteen months before, confirmed by finding T.B. in sputum at time of commencing inoculation, and the rapid disappearance of all symptoms under tuberculin, *no other change in treatment being made.* So far from having faith in the hypodermic needle, every injection was given under protest, to an accompaniment of cries.

**Case 2.**—C. B. First seen September 13th, 1906. A young man of previous good health and physique, actively engaged in business. Three or four weeks before acute pleurisy followed by sign of rapidly extending mischief in right apex. Profuse sweats and high temperature, which had somewhat quieted down when I saw him. There were signs of cavity formation. Sputum and T.B. abundant. General condition good. Opsonic index on two successive days, 1.22 and 1.80; reduced by the first small inoculation to 0.52 in twenty-four hours, but recovering in a few days to 1.50. During this short negative phase an increase in the symptoms was observed, but an improvement then set in which continued throughout the subsequent course of inoculations with little or no interruption. The dose of tuberculin used on the first occasion was 1/2500 mg., and it is probable that a smaller dose would have been preferable. Seven inoculations were given at intervals of about three weeks, and fifteen indices were determined in connection with them. The patient was always very sensitive to tuberculin, the injections producing sharp falls in the index, succeeded by moderate rises of short duration, though in one instance an almost immediate rise was observed. On February 7th, 1907, T.B. had disappeared from the sputum, the opsonic index was 1.50, and heated serum index 0.39, the patient was up and about with a normal temperature, and feeling comparatively well. He was then sent to Torquay for change, and by June was still better. The index on May 10th, 1907, was 1.17, and on June 30th, 1907, 1.00. He increased in weight from 9 st. to 13 st. and the cough and expectoration disappeared, but there were still signs of consolidation, and some moist sounds audible in right apex. At present, a year later, there is no relapse. He has gone to Australia, and sends home encouraging reports. This gentleman was a patient of Drs. Lees and Holmes, and was seen by me once only. It was not possible to get clinical records complete enough to trace the effects of individual doses of tuberculin on temperature, weight and pulse. He was seen in consultation by Dr. Shingleton Smith at an early stage of his illness, and his condition then necessitated the gravest possible prognosis. The evidence the case affords for the usefulness of tuberculin lies in the improbability of such a remarkable improvement under ordinary treatment, and in the consensus of opinion—of the physicians, the patient, and myself—that improvement really followed the inoculations. The case was treated in an ordinary bedroom at home, with, for the first few weeks, absolute rest in bed, a strictly milk diet, and the administration of calcium chloride. Dr. Lees writes me:—"When opsonic treatment was commenced, the patient's condition was most grave, and he was going downhill as rapidly as possible. Dr. Shingleton Smith considered him almost hopeless, and the improvement was truly remarkable."

**Case 3.**—O. M., æt. 20. First seen March 13th, 1907. Strong young man. Designer in father's office. Weight in health, 10 st. 7 lb. Bronchial cough in October, 1905. Lungs examined by Dr. Edgeworth, and found sound. In good health then until January, 1907. January 21st, 1907: Dr. Herapath called in for what appeared to be an attack of influenza. January 31st: temperature continuing and lung trouble being suspected, Dr. Charles called in consultation. Sputum sent to Clin. Res. Ass. and T.B. found. February 12th: temperature and other grave symptoms continuing, Dr. Charles again consulted. Found fluid in chest and aspirated; also took sample of sputum. Examination of both negative as to tuberculosis. February 13th: Mr. Carwardine operated and withdrew 27 oz. cloudy and blood-stained fluid from pleural cavity. Temperature remained up after the tapping, and did not begin to fall until the first inoculation on March 17th, over a month later. Previously, I believe, another sample of sputum had been examined in London, and T.B. found. On March 13th I saw him and examined the sputum, finding T.B. in not great abundance. The opsonic index was 1.41, and there was marked dulness of upper half of left lung. Dr. Markham Skerritt was called in on March 16th to sanction the inoculations which I proposed. This he did, with the reservation that personally he would prefer sending the patient to a sanatorium. I gave the first inoculation on March 17th, and saw him again on March 25th; after that not until he came to Bath to see me on May 1st nearly well. Five inoculations in all were given, and the temperature, which had remained up for over a month after the drainage of the pleural cavity, commenced to fall immediately after the first injection (see chart), and fell regularly with each succeeding one until it became normal. Between the fourth and fifth injections the patient got up and about, and had sub-normal temperatures in the morning. The sputum was scanty throughout, and ceased entirely before the fourth injection. The pulse kept up, 104 to 120, weeks after the temperature had become normal. In May, 1907, he went into the country for a few months, and I saw him again on September 18th in first-rate health, weight 10 st. 12 lb., lungs apparently quite sound, hardly any discoverable difference between the two sides. He resumed work. Last week I heard from his friends that he is as well as ever. The only use that could be made of the opsonic index in this case was to ascertain that each dose of tuberculin did not produce too great a temporary fall, and that there was a recovery before the succeeding inoculation. The fourth injection showed a normal index before and a few days after, and on the complete subsidence of symptoms (except accelerated pulse), the index rose to 2.0. When again tested, five months later, it was normal.

Those who would explain the fall of temperature and progress of this case to recovery apart from the inoculations, must fall back either on the long arm of coincidence or the pleasures of hope. The possibility, but not the probability, of the first is admitted, but the argument loses weight with every succeeding case to which it is applied. As to the second, if admitted it would point to the advantage of injecting hope hypodermically along with tuberculin, thus laying stress on the immaterial, and reversing the famous advice of Voltaire, that if you wish to destroy a flock of sheep with incantations you should administer a suitable quantity of arsenic with them.

**Case 4.**—J. B. E., æt. 19½. Post Office employé. First seen May 29th, 1907. Nearly two and a half years before (Christmas, 1905) nasal catarrh, sore throat and cough. Under treatment by local doctor until March, 1906, when Dr. Markham Skerritt saw him in consultation, diagnosed phthisis, and gave a grave prognosis. Gave up work, and took to open air and milk. Had five weeks on a farm, and then passed his days in a hut which his father had built for him in the garden. March, 1906: weighed 8 st. 1 lb., probably never more than 8 st. 3 or 4 lb. Three weeks at Weston in August, 1906, during which lost 5 lb. in weight. Condition May 29th, 1907 : Carries out the treatment with great strictness, but cannot increase weight even with forced feeding. Frequently vomits ; has to walk with extreme slowness, supported. Exertion of getting into bed often brings on nausea or vertigo. Pulse 144, very weak. Nearly fainted during two or three of the early visits to me. Crepitations posteriorly over upper half of left and upper third of right lung, and in front at left apex. Whispering pectoriloquy near vertebral end of right scapular spine. Night sweats up to November, 1906. T.B. very abundant in sputum. I consented to try tuberculin in this case admittedly as a last resort, for the patient, an exceedingly intelligent youth, had rigidly carried out the best advice obtainable, and was practically under sanatorium treatment, with the additional advantage of a mother's care, and yet was losing ground. The parents were advised to discontinue sending him to me unless speedy improvement should become manifest, and considering the condition of his pulse, a sudden termination of the case during the first weeks would not have surprised me. He has now been under treatment a year, with the results depicted in the chart. Although recovery cannot even now be said to be probable, the difference in his condition is so marked that I consider the case the most interesting of the series. The first inoculations resulted in a gain of 5 lb. in weight in a fortnight, and as he had carefully kept his weight chart for eight months previously, the point is a crucial one. The gain in weight has, on the whole, continued to be progressive, and he now stands at about the highest level yet reached. The temperature chart is even more satisfactory. To save space, the daily range is marked by vertical lines, and it will be seen that for some months past it has differed little from that of a normal man. The pulse has come down more slowly (as in other cases), but during the last few weeks has undergone remarkable improvement, simultaneously with what appears to be a rapid clearing up of the right lung. Commencing with a weekly average of 135 on two readings daily (at 10 and 6), it is now 109 on a weekly average of three readings daily (8 in bed, 10 up, and 6). The break in the pulse chart marks where the three readings system begins. Digitalis was given for a fortnight at an early stage, with a decided reduction in pulse rate, but it was badly borne, and, being open to other objections, was discontinued. Iron was given from the commencement, and the hæmoglobin has risen from 80 to 95 per cent. If it should be thought that iron is responsible for the initial crucial gain in weight, one has only to turn to the chart of the next case, where a much greater gain in weight occurred before iron was given. It was only practicable to determine the opsonic index once a fortnight, viz. at the moment of inoculation. Remembering that the journey to Bath, from some miles beyond Bristol, was the one bit of exercise of the fortnight, and was accomplished with difficulty, the use made of these testings was to be sure that the opsonic power was not depressed to a dangerous point before each injection. It would have been preferable to give the injections at his home, but no one on the spot would undertake it, and a six-cylinder motor-car, which would in this case have possessed decided therapeutic value, was not at my command. As the chart shows, the indices were almost always below normal, but only on two occasions were they too low. Each occasion is significant. The one on July 13th preceded the most serious set-back of the whole time (for which the extreme heat and the rather large inoculation must divide the responsibility), and that of February preceded another period of difficulty, coincident in time with the all-prevailing influenza, which seriously affected other members of the household. During this period the patient lost a little weight, and complained of pain in the right chest. After the passing of the influenza wave he rallied, in common with four other phthisical patients who were similarly and simultaneously affected, and has since progressed to the best condition yet attained. A minor set-back occurred in this and other cases during the very cold snap at the end of December. A timely doubling of all underclothing prevents loss of weight from this waste of heat by radiation solely, in patients of feeble assimilative capacity.

**Case 5.**—W. L., æt. 21. Telegraph engineer. First seen August 1st, 1907. Three and a half years ago had what was called influenza, in Rosario. Removed to Buenos Ayres, where he had a bad hemorrhage; none since. Came home, abundant T.B. in sputum. Remained home fourteen months doing open-air treatment, gained weight, and got better; T.B. disappeared from sputum. Went out again, but got relapse; was to have been sent to Cordoba, which is considered good for consumptives, but was sent to Mendoza instead. Became permanently deaf on left side after large doses of quinine. There got laryngitis and bronchitis, and came home again in June, 1907. Is still hoarse, with continual throat cough. Upper half right lung has increased vocal fremitus and resonance, with dry crepitations and dulness. The temperature seldom rises above 99, and is often 97–98. Has been losing flesh, and now weighs 7 st. 6 lb. The first inoculation was given on August 1st, and on August 27th he had gained 12 lb. in weight (see chart) and felt stronger, with diminshed cough and expectoration. The gain in weight continued, with intervals of marking time, but no relapse until February 17th, when influenza broke out in the household, and the patient, for the first time, developed a temperature of 100.5, and began to lose a little weight. Simultaneously the base of the right lung showed signs of consolidation, but T.B. disappeared from the sputum. Before this set-back the patient had every prospect of a quick cure; he felt strong, and could walk six miles without fatigue. After remaining weak for several weeks he has again picked up his weight and gained strength, but considering that a much larger area of lung is now involved, the prospect is more doubtful. This case allowed of much more exercise than the preceding, and of larger doses of tuberculin. The indices, taken always after journeys to Bath, are hardly ever below normal. Note that on November 18th, after the weight had remained stationary for over a month, an increased dose of tuberculin inaugurated a further rise.

**Case 6.**—L. C. First seen November 2nd, 1907. This young lady had rales in right apex, with slight temperature and T.B. in sputum nearly seven years ago. At once sent to a sanatorium for eight months. Much improved; 7 st. 12 lb. on entering, 9 st. 10 lb. on leaving (13 lb. were gained in first fourteen days on forced feeding). Temperature normal or sub-normal on leaving; 99–100 on entering. Still a little cough, and some T.B. in sputum. Soon had a relapse, and has been gradually getting worse, with considerable intervals of improvement. Open-air treatment followed throughout very intelligently and perseveringly. Was also some months at an Austrian cure. Slight hæmoptysis on several occasions. November, 1906, signs of a large cavity in right upper lobe. Again in sanatorium for nine months, during which time cavity apparently diminished in size. Weight rose from 7 st. 1 lb. to 7 st. 12 lb. in three months, but declined again to 7 st. 5 lb. in next six months, when opsonic treatment at home was advised by Dr. Fraser, instead of a return to a sanatorium. November 2nd, 1907: T.B. very numerous; coagulation time, 3 min. 30 sec.; frequent epistaxis; hæmoglobin, 80 per cent.; signs of cavity under sternal half of right clavicle about size of a five shilling piece; moist sounds all over upper half of right lung at times, but they frequently disappear almost entirely. First sanatorium spoiled the appetite, which is now feeble and capricious, and any little gain in weight is soon lost. The weight chart for several months before inoculation is appended. There is again to be noted a crucial gain in weight directly the inoculations were commenced, continuing, with some variations, until the height of the influenza wave. The patient had on the whole made undoubted progress, when on January 25th she assisted in entertaining visitors at a crowded "At Home" (many of the visitors coming from influenza-infected households), complained of great fatigue, and almost at once took to bed with relapse in all symptoms. Up to this time exercise had been severely restricted, milk and lime water had been freely given, also calcium chloride (with the result of stopping the attacks of epistaxis), and the inoculations had been of average amount and at rather long intervals. There were unusual opportunities of studying the opsonic aspects of this case, and a great number of indices was taken, enabling one to make a probable curve (see chart). The index was before inoculation subject to great fluctuation, and affords a good example of what happens in these cases of systemic infection. Any three or four successive indices selected from the chart would afford good evidence of infection in a case where the diagnosis was doubtful. It is plain that the effects of individual inoculations are obscured by these natural fluctuations, and however many indices are taken, they do not serve as a guide to individual inoculations. But, on the whole, it is readily seen that no negative phase of even a day's duration has been encountered during the whole course of inoculations, for there are few indices below normal, and every one of them is succeeded by a much higher one on the very next occasion of testing. Wishing to obtain a better control, I kept the patient in bed fifteen days during her relapse, on milk and calcium chloride, and waited to see if the temperature would subside without inoculation. As it showed little tendency to do so, I gave a tiny experimental injection on the afternoon of February 22nd, and this was followed by an immediate drop in temperature and corresponding rise in the opsonic index. Another, on February 27th, produced a similar result, and since then the temperature has been more nearly normal than at any time before. Under the system of small inoculations frequently repeated, she has recovered ground quickly, gaining weight at the

**Charts of Cases 3, 4, 5 and 6.**—The original chart was drawn on section paper, but in consequence of the section lines appearing too prominent in the block, a tracing was taken and a new block made.

In all the charts inoculations are shown by arrows, the length being proportional to the dose.

Since May 26th Case 6 has gained another 4 lb., and Case 4's pulse-rate is now below 107 weekly average. Case 5 has regained much of the strength lost in March, and again takes 6-mile walks. (June 16th, 1908.)

rate of 1 lb. per week for ten weeks, and now stands at record weight for four years, and feels correspondingly better The chest signs are better than at any time since inoculations were started. It is noteworthy that the opsonic index has been much more consistently high under this plan, which appears to suit this patient admirably. The activity of the patient's leucocytes was tested on one occasion by Veitch's plan for taking the hæmo-phagocytic index, and found to be good. Except for this purpose, the method seems to offer no advantage to one in the habit of regular opsonic work. On another occasion the dilution method was used, but it brought out no very great difference between the patient's and normal blood, and could seldom be used on account of the vastly greater labour.

**Case 7.**—C. E. P., æt. 34, tailor. First seen December 21st, 1907. Two months' cough with expectoration, but since fifteen years old subject to hoarseness and impaired voice on slight cold or over-exertion. T.B. had been found in sputum. Advised by doctor, on diagnosis of right apical phthisis, to give up business and go abroad to a sanatorium. Impossible. Applied for admission at Winsley ; after an interval accepted, but no bed vacant. Feels as strong as ever. Sleeps well, but not so well owing to cough. Has only lost 2 lb. in two years—now 8 st. 10 lb. Appetite good. Two night sweats recently. No hæmoptysis or diarrhœa. Voice weak and hoarse. Pulse, 68 ; temperature, 97.2 ; hæmoglobin, 95 per cent. Some dulness right and left apices front. No rales. Breath sounds almost normal. Inspiration, 32 in. ; expiration, 30 in. Foul sputum, with many T.B., long streptococci and staphylococci. Opsonic index, 2.0. First inoculation made him feel worse for two days, then suddenly better. After three inoculations did not feel that there was anything to consult me for. Voice natural, better than for two years past ; sputum less than half, but T.B. abundant. By March 18th had increased weight to 9 st. 2½ lb., fell on April 22nd to 9 st., since increased to 9 st. 3 lb. on May 6th. The busy time and the influenza weather have tried him lately. Here is a case running an almost normal temperature, with normal pulse, no considerable loss of weight or loss of appetite or strength ; in fact, nothing *chartable* on which to found a decided judgment as to the action of tuberculin. Opsonic indices were taken regularly every fortnight, but after the first highly significant reading (2.0) have only varied between .80 and 1.22. As to chest signs, on first examining him, I felt somewhat in the position of the aged and slightly deaf consultant, who was called in to a case of pneumonia, and whispered to the family doctor on the stairs, "Which side is it ?" As I had given the sputum to my assistant to stain before commencing the examination, and inspected the T.B. before its conclusion, I was able to speak with certainty as to the diagnosis, in spite of the almost total absence of physical signs. The increase in weight after inoculation, though adding force to the similar cases, is hardly crucial, but the unmistakable improvement after three inoculations may be considered crucial, though not chartable.

If tuberculin can show distinct evidence of specific action in serious, acute and longstanding cases of phthisis like the few here cited, cannot much more be expected of it in very early and slight cases ? Where the diagnosis is clearly established, it seems to me it should be the very first means used after the patient has been placed under suitable conditions; and as the essentials of rest, fresh air, and good feeding can generally be obtained outside a sanatorium, I believe that tuberculin without a sanatorium can do more than a sanatorium without tuberculin.

Finally, there are the very numerous cases of children and young people beginning to ail who ought to be in perfect health. The family doctor suspects a tubercular tendency, or "delicacy of chest," but no unimpeachable symptoms are discoverable. If, however, there is a group of suspicious little facts, explicable by incipient tuberculosis though not diagnostic of it, and if a series of three or four opsonic indices (two of which may advantageously be taken before and after exercise respectively) shows the fluctuations so often met with in actual infections, it will probably be found that a few inoculations will at once establish the diagnosis, and cure the patient. Several cures of this kind have come under my observation, during the two years I have acted on the obvious indication.

### CALMETTE'S OPHTHALMIC REACTION IN TUBERCULOSIS.*

BY

J. M. Fortescue-Brickdale, M.A., M.D. Oxon.,

## Figures and Tables

**Figure f1:**